# Color Development Characteristic and Kinetic Modeling of Maillard Reaction in Membrane-Clarified Sugarcane Juice During Vacuum Evaporation Process

**DOI:** 10.3390/foods14122136

**Published:** 2025-06-19

**Authors:** Meiyi Han, Hongkui Zhao, Zhihua Liu, Jing Liu, Xi Liu, Fangxue Hang, Kai Li, Caifeng Xie

**Affiliations:** 1 College of Light Industry and Food Engineering, Guangxi University, Nanning 530004, China; 2116391008@st.gxu.edu.cn (M.H.); 2216301064@st.gxu.edu.cn (H.Z.); lzh666@st.gxu.edu.cn (Z.L.); 2116391020@st.gxu.edu.cn (J.L.); 2216391026@st.gxu.edu.cn (X.L.); hangfx@163.com (F.H.); gxlikai@gxu.edu.cn (K.L.); 2 Provincial and Ministerial Collaborative Innovation Center for Sugar Industry, Nanning 530004, China; 3 Engineering Research Centre for Sugar Industry and Comprehensive Utilization, Ministry of Education, Nanning 530004, China

**Keywords:** sucrose, ceramic membrane filtration, Maillard reaction, melanoidins

## Abstract

This study systematically investigated the evolution of color values and the reaction kinetics of the Maillard reaction in membrane-clarified sugarcane juice during the vacuum evaporation process, providing a theoretical basis for pigment regulation in white sugar production. Content changes in the reactants (sucrose, glucose, fructose, and free amino acids), the precursors of melanoidins including 3-deoxyglucosone, 5-hydroxymethylfurfural, glyoxal, methylglyoxal, carboxymethyl lysine, and melanoidin, were monitored during the thermal processing of membrane-clarified sugarcane juice (MCSJ), and the reaction mechanism was investigated via kinetic modeling. The zero-level, first-level, and second-level kinetic models could represent the change in L* and b*, and the zero-level kinetic model best fit the change in a* and ΔE*. The multi-response kinetics revealed that the main pathway of melanoidins in MCSJ model systems was that glucose and fructose were mutually isomerized into 1,2-enediol to generate 3-DG and then degraded to produce 5-HMF. Subsequently, 5-HMF further reacted to produce melanoidins.

## 1. Introduction

Sucrose, the world’s leading sugar substance, is a natural sweetener, with nearly 80% being produced from sugarcane. The mixed sugarcane juice in sugarcane factories contains various solid particles (sand and bagacillo), colloidal materials (pectin, protein, starch, etc.), and soluble non-sugar substances (polyphenols, organic acids, inorganic salts, etc.) with a concentration of 12–16°Bx. Therefore, it has to be clarified [1,2] and then sent to the evaporation process and the boiling process for white sugar production.

China, the world’s fourth-largest producer of sucrose, with an annual sucrose output of 10 to 14 million tons, traditionally employs the sulfitation process for sugarcane juice clarification. However, with the increase in consumers’ health awareness, white granulated sugar produced using the sulfitation process no longer satisfies consumers due to the high sulfur dioxide residue content, so a new clarification method, namely, ceramic membrane clarification technology with no chemical agents, has been applied in sugar factories in China [3]. Amino acids and reducing sugars are both water-soluble and can pass through the ceramic membrane; then, they remain in the membrane-clarified sugarcane juice (MCSJ), where they are susceptible to the Maillard reaction (MR) during the subsequent evaporation and boiling processes and then produce melanoidins, resulting in an increase in color of the finished white granulated sugar [4,5], which limits its application in high-end food and beverages. Hence, it is imperative to investigate the color development and kinetics of the MR during heat treatment in MCSJ to provide theoretical guidance for developing color-controlling technologies for white granulated sugar production.

Indeed, it is widely recognized that the MR exhibits a strong correlation with a multitude of factors, encompassing the chemical composition of the reactants, the reaction pH and time, and the heating method and temperature [6,7], as well as the types and contents of co-existing substances such as polyphenols, inorganic ions, etc. Therefore, the MR of MCSJ must be quite different from the MRs detailed in previous reports, such as those used for Japanese squid [6], orange juice [8], grape juice [9], etc. Hemmler et al. (2018) found that the products generated by the MR between ribose and four different amino acids were significantly different in composition and content [10]. Asikin et al. (2015) also indicated that the heating temperature significantly affected the levels of MR products, particularly pyrazine and furan, in syrup [11]. Liu et al. (2022) observed that the increase in pH significantly enhanced the MR of sugarcane juice during the evaporation process at 150 °C [12]. However, studies on the MR occurring in sugarcane juice during thermal treatment mainly focused on the characteristics and identification of volatile substances [12,13,14] and the formation and kinetics of harmful compounds [15], whereas the color-enhancing properties and their detailed kinetic mechanisms have not been clarified yet.

The objective of this study was to elucidate the color development characteristics of the MR in MCSJ via quantitative analysis of the contents of the substrates and key products of the MR in MCSJ and model systems consisting of three dominant amino acids and sugars under vacuum thermal treatment. The kinetics of the MR in MCSJ was studied by using a simple kinetic model and the multiple-response model, and the main pathways of the MR during vacuum evaporation were proposed.

## 2. Materials and Methods

### 2.1. Materials and Chemicals

Mixed sugarcane juice was provided by Guangxi Mingyang Sugar Factory, China. A ceramic ultra-filtration membrane (50 nm) was provided by the Institute of Membrane Science and Technology of Nanjing University of Technology. Fructose, glucose, and sucrose were purchased from Sigma Aldrich (St. Louis, MO, USA). Lysine, histidine, proline, glyoxal (GO), and methyl glyoxal (MGO) were purchased from Macklin (Shanghai McLean Biochemical Technology Co., Ltd., Shanghai, China). 3-deoxyglucosaldoketone (3-DG) and 5-hydroxymethylfurfural (5-HMF) standards were purchased from Shanghai Yuanye Bio-Technology Co., Ltd. (Shanghai yuanye Bio-Technology Co., Ltd., Shanghai, China). All the reagents were of analytical grade. A Nε-carboxymethyllysine (CML) ELISA Kit and a Nε-carboxyethyllysine (CEL) ELISA Kit were obtained from Shanghai Enzyme-linked Biotechnology Co., Ltd., (Shanghai Enzyme-linked Biotechnology Co., Ltd., Shanghai, China).

### 2.2. Sample Preparation

The mixed juice was filtered by a stainless steel mesh with a pore size of 100 mesh, then heated to 90 ± 1 °C and filtered by a 50 nm ceramic membrane. The resulting MCSJ was evaporated in a steam-heated stainless steel evaporation tank (ENCO-JM1, Hangzhou Enchuang Machinery Co., Ltd., Hangzhou, China) under a vacuum of 0.08 MPa until it was concentrated to 60–66°Bx, followed by immediate ice-bath cooling post-thermal treatment. The samples were taken at intervals for analysis during the evaporation process.

### 2.3. Preparation of MCSJ Model Systems

To investigate the kinetics and primary pathways of the MR in MCSJ during heating treatment, the MCSJ model systems comprising three sugars (sucrose, fructose, and glucose) and three amino acids (lysine, histidine, and proline) were prepared. Lysine, histidine, and proline were chosen as the dominant amino acids of MCSJ because of their higher content, higher consumption rate, and high correlation with the amount of melanoidins in membrane-clarified juice during heat treatment, according to previous studies [16]. The model system was formulated by accurately metering and blending refined sucrose, fructose, glucose, and selected amino acids, and the specific composition of the model system is shown in Table 1. The mixture was dissolved in sodium acetate buffer solution (0.07 M sodium acetate and 0.03 M acetic acid) to attain a 15°Bx concentration, subsequently heated and evaporated under vacuum (0.08 MPa) at 90 °C until reaching a final concentration of 60–66°Bx, and then rapidly quenched in an ice bath. During thermal treatment, samples were periodically analyzed in triplicate.

### 2.4. Determination of Colorimetric Parameters

The L*, a*, and b* values of the samples were measured using a Color Quest colorimeter (CM-3600d, Konica Minolta, Japan), as described by Farroni et al. (2012) [17]. The total color difference (ΔE*) was computed using the following Equation (1):

(1) $$∆E*=\sqrt{{\left(L^{*}-{L_{0}}^{*}\right)}^{2}+{\left(a^{*}-{a_{0}}^{*}\right)}^{2}+{\left(b^{*}-{b_{0}}^{*}\right)}^{2}}$$

L*, a*, and b* represent the lightness, greenness/redness, and blueness/yellowness of samples during the evaporation, respectively; L_0_*, a_0_*, and b_0_* represent the lightness, greenness/redness, and blueness/yellowness of the samples before evaporation; ΔE* represents the total color difference. Perceivable color differences can be categorized as follows: not noticeable (0–0.5), slightly noticeable (0.5–1.5), noticeable (1.5–3.0), and highly visible (3.0–6.0), as outlined by Limbo et al. [18].

### 2.5. Determination of Sugars

The contents of sucrose, fructose, and glucose were assayed by adopting and modifying the methodology outlined by Yi et al. (2017) [19]. After appropriate dilution, the samples were filtered through a 0.22 µm membrane. Gel permeation chromatography (AGILENT 1260, Agilent Technologies, Palo Alto, CA, USA) was then performed using a ZORBAX Original 70 Å NH_2_ column (Φ4.6 mm × 250 mm, 5 µm, Agilent Technologies, USA) with acetonitrile–water (75:25 by volume) as the mobile phase, a differential refractive index detector, a column temperature of 40 °C, an injection volume of 10 µL, and a flow rate of 1.0 mL/min.

### 2.6. Determination of Protein and Amino Acids

The determination of protein content was conducted using a method based on Bradford’s protocol, with slight modifications, as outlined by Hildebrandt et al. (2008) [20] and detailed below: Initially, 100 mg of Coomassie Brilliant Blue G-250 was accurately dispensed into a 1000 mL brown volumetric flask. Then, 50 mL of 95% ethanol and 100 mL of 85% phosphoric acid were sequentially added, followed by deionized water to the 1000 mL mark. One milliliter of each sample (diluted to approximately 15°Bx) was taken and subsequently transferred to 10 mL test tubes, where 5 mL of Coomassie Brilliant Blue G-250 solution was added to each. After allowing the mixture to stand for 10 min, the absorbance at 595 nm was measured using a Microplate Reader (Infinite E Plex, Shanghai, China). The protein standard solution was prepared with bovine serum albumin, and a standard curve depicting the correlation between protein concentration and absorbance was subsequently established. Based on this curve, the protein content of the sample was determined.

The amino acid composition was ascertained utilizing the automatic amino acid analyzer, L-8900 (L-8900, Tokyo, Japan), according to the methodology outlined by Triki et al. (2018) [21].

### 2.7. Determination of Key MR Products

#### 2.7.1. Determination of α-Dicarbonyl Compounds

MGO and GO were determined using gel permeation chromatography (AGILENT 1260, Agilent Technologies, Palo Alto, CA, USA) according to the method outlined by Li et al. (2022) [22]. After centrifuging the sample at 10,000 rpm for 10 min, 2 mL of the supernatant was combined with 1 mL of 0.6% o-phenylenediamine solution and 150 µL of 0.5 mol/L phosphate buffer (pH 8.0). The mixture was subsequently shielded from light and incubated at 60 °C in a water bath for 4 h. Subsequently, the solution was filtered using a 0.22 μm microporous filter membrane and transferred into a sample bottle for further analysis. Detector: UV detector. The mobile phase comprised a mixture of 0.1% formic acid and acetonitrile (75:25, *v*/*v*). It was eluted using an Agilent ZORBAX SB-C18 column (manufactured in Santa Clara, CA, USA) at a flow rate of 0.6 mL/min. The column oven was maintained at 25 °C, and detection was performed at a wavelength of 314 nm.

The quantification of 3-DG was conducted according to the method described by Asikin et al. (2013) [23], with minor modifications. A total of 2 mL of the sample was mixed with 1 mL 1.6 mmol/L of 2,3-diaminaphthalene, then incubated in a water bath at 50 °C for 24 h, filtered through a 0.22 µm microporous membrane, and transferred to a sample bottle for gel permeation chromatography (AGILENT 1260, Agilent Technologies, Palo Alto, CA, USA) analysis. Detector: UV detector. The mobile phase consisted of methanol (0.1% formic acid): water (40:60, *v*/*v*), with an Agilent ZORBAX SB-C18 (Santa Clara, CA, USA), with a flow rate of 0.8 mL/min. The column oven temperature was 40°C, and detection was performed at a wavelength of 268 nm.

#### 2.7.2. Determination of 5-HMF

The concentration of 5-HMF was assayed using the methodology outlined by Polokova et al. (2017) [24], with certain modifications applied. A 4 g sample was combined with 0.3 mL of 15% potassium ferrocyanide and 0.3 mL of 20% zinc sulfate in a 10 mL volumetric flask, followed by dilution to the mark with ultrapure water. After allowing the mixture to stand for 10 min, it was filtered through a 0.22-micrometer microporous membrane filter, and the resultant filtrate was dispensed into a sample vial for gel permeation chromatography (AGILENT 1260, Agilent Technologies, Palo Alto, CA, USA) analysis. Detector: UV detector. The mobile phase (80:20, MeOH: H_2_O, *v*/*v*) was eluted using an Agilent ZORBAX SB-C18 column (Santa Clara, CA, USA) at 0.7 mL/min. The column oven temperature was 40 °C, and detection was at 284 nm.

#### 2.7.3. Determination of CML and CEL

CML and CEL were using ELISA quantified kits (Shanghai Enzyme-linked Biotechnology Co., Ltd., Shanghai, China) based on Ojeda’s method [25].

#### 2.7.4. Determination of Melanoidins

Melanoidins were quantified based on Xiao’s method [26] with minor modifications. L-aspartic acid and glucose were dissolved in DI water, incubated at 90 °C for 10 h, filtered through a 0.45 µm membrane, dialyzed, and lyophilized to yield the standard. A calibration curve depicting the relationship between melanoidin concentration and absorbance was established. The absorbance of samples at 420 nm was measured using a UV-visible spectrophotometer (UV-2501PC, Shimadzu, Kyoto, Japan), with the measurements repeated three times. The melanoidin concentration in the samples was assessed utilizing the predefined standard curve.

### 2.8. Kinetic Modeling

The change in key MR products of model systems at different temperatures (60 °C, 70 °C, 80 °C, and 90 °C) was modeled by using simple kinetic models [27], including zero-level kinetic model (Equation (2)), first-level kinetics model (Equation (3)), and second-level kinetics model (Equation (4)). Arrhenius analysis (Equation (5)) was employed to compute the apparent activation energy (E_a_).

(2) $$C=C_{0}+k_{0}t$$

(3) $$C=C_{0}exp(k_{1}t)$$

(4) $$\frac{1}{C}-\frac{1}{C_{0}}=k_{2}t$$

(5) $$k=Aexp[-\frac{-Ea}{RT}]$$

where C represents the values of the measured indicators in the model system at time t; k_0_, k_1_, and k_2_ represent the reaction rate constants of zero-level kinetics, first-level kinetics, and second-level kinetics, respectively. E_a_ is the activation energy (kJ∙mol^−1^), R is the universal gas constant (8.314 J∙mol^−1^∙K^−1^), T is the absolute temperature (K), and A is the pre-exponential factor.

Furthermore, we utilized the multiple-response model to determine the reaction mechanism and identify the pivotal steps leading to the formation of key MR products in MCSJ. This model enabled us to comprehensively understand the entire reaction mechanism and facilitated the quantitative description and prediction of changes occurring at specific temperature-time points. Athena Visual Studio software, version 21.1 (Athena Visual Software, Inc., Naperville, IL, USA), was utilized for the numerical integration and estimation of the model parameters.

### 2.9. Statistical Analysis

Statistical significance of mean differences was assessed using one-way ANOVA, followed by Tukey’s multiple comparison test, using a *p* < 0.05 threshold for significance. Triplicate experiments were conducted for each assessment.

## 3. Results and Discussion

### 3.1. Changes of Colorimetric Parameters of MCSJ and Model System

**Figure 1:**
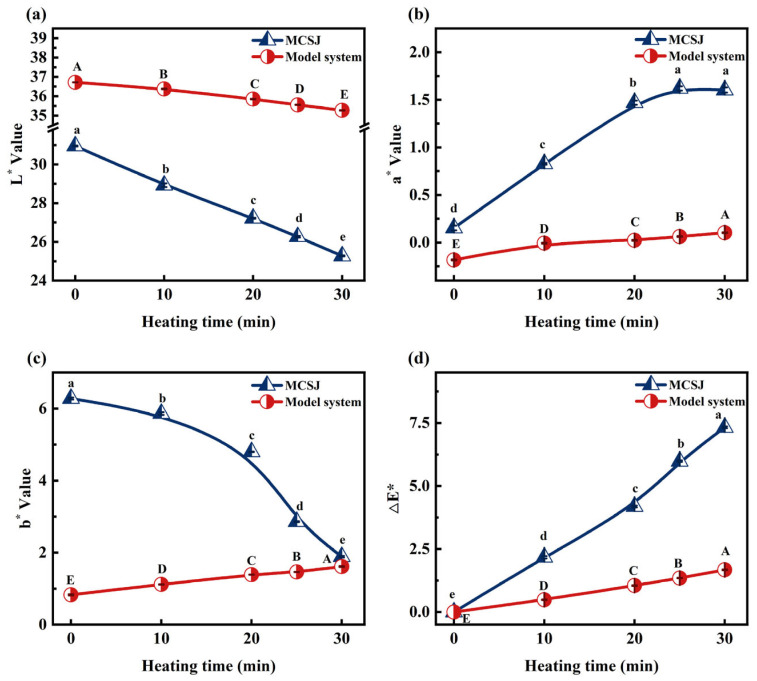
Colorimetric parameters of MCSJ and model system, (**a**) L*, (**b**) a*, (**c**) b*, and (**d**) ΔE*, during vacuum evaporation. Lowercase letters represent significant differences (*p* < 0.05) between samples with different heating times in the MCSJ system; uppercase letters represent significant differences (*p* < 0.05) between samples with different heating times in the model system.

Before heating, the pH values of MCSJ and the model system at room temperature were 6.42 and 6.5, respectively. After heating, the temperatures reached 90.0 ± 2 °C and 89.5 ± 2 °C, respectively. The colorimetric parameters of MCSJ and the model systems during vacuum evaporation are shown in Figure 1. Increased treatment time significantly reduced L* in both reaction systems and b* in MCSJ (*p* < 0.05) while significantly growing a* and ΔE* in both MCSJ and the model systems (*p* < 0.05). This indicates the darkening and reddening of samples, attributed to Maillard reaction-induced melanoidin formation between reducing sugars and amino acids. The longer the reaction time, the more melanoidins were produced. As a result, the ΔE* of MCSJ and the model system increased remarkably. When the treatment times exceeded 10 and 30 min, the color of MCSJ and the model system became noticeable. It is noteworthy that the ΔE* value of MCSJ significantly exceeded that of the model system, suggesting a markedly higher rate of melanoidin production via the MR in MCSJ compared to the model system, alternatively indicating that polyphenols in MCSJ underwent a coloring reaction.

During the evaporation process, the trend of b* in MCSJ and model systems was opposite, i.e., the b* value of MCSJ markedly decreased (*p* < 0.05); in contrast, the model systems markedly increased (*p* < 0.05). However, the b* of MCSJ was much higher than that of the model system. This might be because MCSJ also contained various other pigments and pigment precursors, such as lutein, polyphenols, ions, etc. [28], and they also underwent oxidation, complexation, degradation or inhibited the MR during the evaporation process, resulting in a more significant decrease in b* than the increase in b* caused by the MR. Therefore, the blue color of MCSJ increased, while the model system without other pigments or pigment precursors turned yellow, whose color entirely relied on the MR. Except for the b* value, the color value changes of MCSJ and the model system follow a similar trend, indicating that the simulated system we constructed is comparable to the membrane-filtered sugarcane juice system in terms of color changes caused by the Maillard reaction. This indirectly supports the rationality of the model we built.

### 3.2. Change in Sugars of MCSJ and Model System

**Figure 2:**
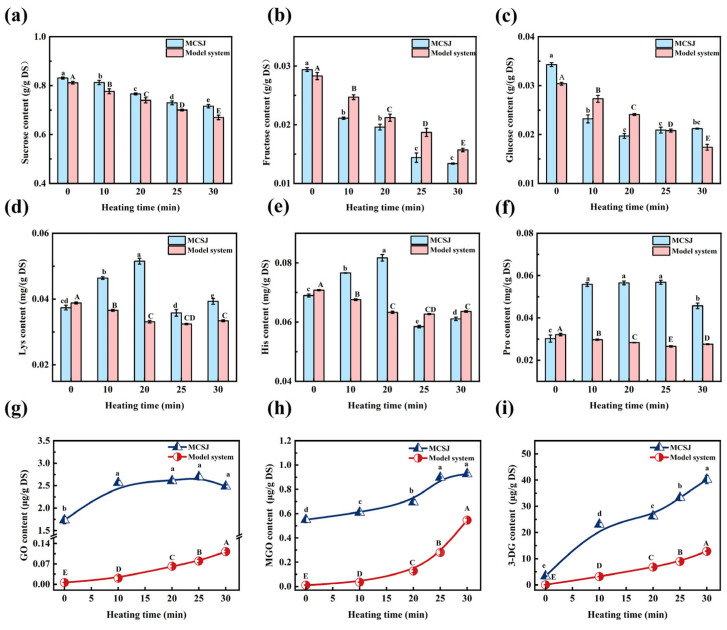
Changes in sugars in MCSJ and model system during vacuum evaporation ((**a**), sucrose; (**b**), fructose; (**c**) glucose; (**d**), Lys; (**e**), His; (**f**), Pro; (**g**), GO; (**h**), MGO; (**i**), 3-DG). Lowercase letters represent significant differences (*p* < 0.05) between samples with different heating times in the MCSJ system; uppercase letters represent significant differences (*p* < 0.05) between samples with different heating times in the model system.

Monitoring sugar consumption is an effective method to assess the extent of the MR [29]. The contents of sucrose and fructose decreased in both MCSJ and model systems during vacuum evaporation (Figure 2a,b). The sucrose content of MCSJ significantly reduced from 0.831 to 0.716 g/g DS at a relatively constant rate, whereas in the model system, it decreased steadily for the first 20 min. This was consistent with the findings of Adulvitayakorn (2019) [30]. Sucrose in MCSJ and model systems does not undergo a direct MR. However, it would hydrolyze into reducing sugars (MCSJ and the model system pH values were 6.42 and 6.50, respectively), which then undergo the MR with amino acids, decreasing the sucrose content [31]. The different trend in the decrease in sucrose content in MCSJ and the model system might be due to the effect of various metal ions (Mg^2+^, Ca^2+^, etc.) on sucrose decomposition in MCSJ [32].

The glucose and fructose content in MCSJ and the model system exhibited a general decreasing trend (Figure 2b,c). In MCSJ, glucose content specifically declined from 0.0343 to 0.0197 g/g DS within the first 20 min, subsequently increasing slightly to 0.0212 g/g DS, resulting in a total consumption rate of 38.19%. However, the contents of glucose and fructose in the model system decreased at a relatively constant rate during the evaporation process. The variations of glucose and fructose content can be ascribed to the combined effects of sucrose decomposition, reducing sugar decomposition, and the MR. The glucose content of MCSJ slightly increased during the late heating period, possibly due to the decrease in pH value of MCSJ-accelerated sucrose decomposition, leading to more glucose being generated than consumed, thus contributing to an increase in glucose content. However, the fructose content showed a continuous decrease with a consumption rate of 54.42% throughout the entire evaporation, indicating that more fructose was consumed by the MR than it generated, which was much higher than that of glucose. This might be due to the fact that the cyclic structure of fructose is prone to ring-opening to generate furanofructose cations (FFCs) and therefore has a high enolization rate, while glucose is structurally stable and has a relatively low rate of enolization. This does not coincide with the previous reports that the reactivity of aldoses is higher than that of ketoses in browning systems, which mainly contributes to the complex chemical compositions of MCSJ [33].

The varying trend in glucose concentration between MCSJ and the model system is attributable to the differing compositions of the two systems. The co-existing phenolic substances and metal ions in MCSJ, such as epicatechin, gallic acid, Ca^2+^, etc., affect the MR [34] and sugar decomposition, resulting in fluctuating amounts of glucose and fructose. In contrast, the model system contained nothing but sugars and amino acids, and the MR rate was stable, leading to a stable decrease in the contents of glucose and fructose. Moreover, MCSJ contains 18 amino acids, and all of them underwent the MR synchronously during the evaporation process, which affected each other [35]; as a result, the MR in MCSJ was much complex.

### 3.3. Changes in Protein and Amino Acids of MCSJ and Model System

The free amino acids are important reactants of the MR. The MCSJ contained 18 free amino acids, totaling 4.8844 mg/g DS (Table 2). It included three alkaline amino acids (lysine, histidine, and arginine), with a combined content of 0.1533 mg/g DS; two acidic amino acids (aspartic acid and glutamic acid), totaling 3.5006 mg/g DS; and thirteen neutral amino acids, amounting to 1.2305 mg/g DS. Notably, the level of acidic amino acids was markedly higher than neutral and alkaline amino acids in concentration. Apart from Gly, Cys, Met, and Phe, all remaining amino acids and the overall amino acid content demonstrated an initial rise, followed by a subsequent fall, culminating in a marginal increase during evaporation. Specifically, the total amount of amino acids in MCSJ increased to 5.7911 mg/g DS from 4.8844 mg/g DS during the first 20 min of evaporation, then decreased to 4.8118 mg/g DS at 25 min and increased again to 5.0261 mg/g DS after the evaporation process. During the evaporation process, most amino acids reached their content inflection points at the 20 min mark, indicating that the amino acids produced by protein decomposition exceeded those consumed by MR during this period, and when the heating time was longer than 20 min, the contents of amino acids began to decrease, indicating the consumption rates of amino acids were higher than the production rate. Fluctuations in amino acid levels indicated variable MR and protein decomposition rates in MCSJ during evaporation. Additionally, the variations in Asp and Glu content in MCSJ may be related to the degradation and Maillard reaction consumption of asparagine (Asn) and glutamine (Gln) [36]. In the first 20 min of heating, the levels of Asp and Glu gradually increase, likely because the decomposition of asparagine and glutamine into Asp and Glu occurs more rapidly than their consumption in Maillard reactions. After 20 min of heating, the decrease in Asp and Glu levels is primarily due to the Maillard reaction, the consumption of asparagine and glutamine, and their degradation. This leads to a situation where the degradation rate of asparagine and glutamine in Asp and Glu is slower than the consumption rate in the Maillard reaction [16,37]. As shown in Figure 2d–f, the concentrations of Lys, His, and Pro in the model systems declined at a constant rate during the first 25 min, then increased slightly in the last 5 min, indicating that the MR occurred at a relatively steady rate. At the end of evaporation, the contents of most amino acids in MCSJ and the specified three amino acids in the model systems exhibited an increase, presumably as a consequence of the liberation of amino acids during the breakdown of Amadori products (ARPs) into α-dicarbonyl compounds [38], which aligns with the observations made by Deng et al. (2021) [39], who documented an upward trend in amino acid liberation from ARPs at pH levels of 5.5 and 7.5, both at 100 °C. This phenomenon could stem from the reduced reactivity of the amino acids upon release or their involvement in the formation of short-chain α-dicarbonyl compounds.

The consumption rates of His, Lys, Arg, and Cys in MCSJ were 21.45–25.21%, much higher than the others, indicating that alkaline amino acids and Cys in MCSJ were prone to undergoing the MR [40]. The initial protein content of MCSJ was 1.319 mg/g DS, and it continued to decrease throughout the evaporation process, with a total consumption rate of 38.36%, which was attributed to the decomposition of protein under high temperature, causing an increase in amino acid content.

### 3.4. Changes in Key MR Products of MCSJ and Model System

#### 3.4.1. Changes in α-Dicarbonyl Compounds

The α-dicarbonyl compounds (α-DCs), including GO, MGO, and 3-DG, serve as crucial reactive intermediates in Maillard reactions (MR), and they are the primary precursors for the formation of melanoidins in thermally processed foods. These compounds are also involved in the formation of Strecker aldehydes and the advanced stages of MR [41], and they are crucial factors contributing to the generation of carboxymethyl lysine (CML) [42].

Variations in the content of α-dicarbonyl compounds in MCSJ and the model system during vacuum evaporation are depicted in Figure 2g–i. The initial concentrations of GO, MGO, and 3-DG in MCSJ were 1.734 mg/g DS, 0.550 mg/g DS, and 3.329 mg/g DS, respectively, which were mainly due to the MR [43] that occurred during the mixed juice heating process before membrane clarification. They continued to undergo the advanced stage of the MR during evaporation. During thermal vacuum evaporation, there was a general augmentation in the concentrations of GO, MGO, and 3-DG within MCSJ, but the increasing trends were not precisely the same. Specifically, the GO content increased rapidly from 1.734 µg/g DS to 2.564 µg/g DS during the first 10 min of heating and then increased slowly to 2.696 µg/g DS from 10 to 25 min and finally decreased to 2.483 µg/g DS during the last 5 min. This is consistent with the findings of İltırak [44] regarding the formation of Maillard reaction products while heating different grains. The main reason was that the MR between reducing sugar and amino acid in MCSJ mainly generated Schiff bases through 1,2-enolization. Then, Schiff bases were rearranged and degraded to form α-DCs, such as 1-deoxyglucosidone (1-DG), 3-DG, and glucuronidone, etc., and then GO was mainly produced by the cleavage of 1-DG and glucuronide as well as by the hydrolysis of Schiff bases. The rapid increase in GO content in the early heating period could be attributed to the rapid hydrolysis of the Schiff base. Concurrently, the formed GO would continue to undergo advanced MR, leading to the production of melanoidins and other compounds (e.g., Strecker degradation products), which consequently caused a decline in the GO content. As a result, the rate of GO content augmentation diminished as the heating duration was extended. When the heating time exceeded 25 min, the GO content tended to decrease because the amount of GO consumed in advanced MR surpassed the amount generated. Moreover, the polyphenols in MCSJ might inhibit MR, resulting in less GO produced [45]. The 3-DG and MGO contents of MCSJ increased throughout the evaporation process. 3-DG, produced via dehydration and hydrolytic deamination of ARP or Heyns compounds, has higher contents than GO and MGO. This aligns with Aktag’s findings [46] that short-chain α-dicarbonyls (e.g., GO, MGO) are less abundant than C6-backbone α-dicarbonyls (e.g., 3-DG) in orange juice and peach nectar. The disparity stems from MR’s preference for forming 3-DG with a C6 backbone [47]. The increase in MGO is consistent with what Feng et al. (2021) reported [48]. MGO is mainly generated by the reverse aldol cleavage of 1-DG and 3-DG. At the same time, MGO continues to react with amino acids to form α-amino ketone, carboxymethyl lysine, and melanoidins, leading to a decrease in content. The increase in 3-DG and MGO content of MCSJ throughout the evaporation process indicated that MR produced more 3-DG and MGO than it consumed.

The concentrations of GO, MGO, and 3-DG in the model system rose at a relatively consistent rate during the evaporation process, which differed from MCSJ, especially for GO and 3-DG. The main reason for this was that the reaction substrates in MCSJ and the model system were quite different. There were 18 amino acids and various co-exciting substances in MCSJ. All the amino acids undergo MR synchronously and interact. Additionally, the co-exciting substances, such as epicatechin and gallic acid, can capture the α-dicarbonyl compounds at the C-6 or C-8 position by their A rings to form adducts, so fewer α-DCs were produced [34,49]. As a result, its MR is much more complex compared to the modeled systems.

#### 3.4.2. Changes in 5-HMF

**Figure 3:**
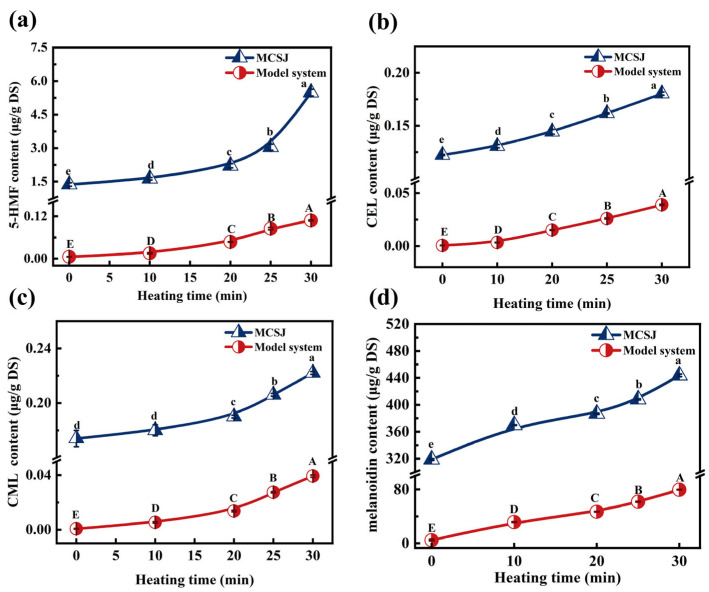
Changes in sugars in MCSJ and model system during vacuum evaporation ((**a**), 5-HMF; (**b**), CEL; (**c**), CML; (**d**), melanoidin). Lowercase letters represent significant differences (*p* < 0.05) between samples with different heating times in the MCSJ system; uppercase letters represent significant differences (*p* < 0.05) between samples with different heating times in the model system.

5-HMF is an essential precursor of melanoidins of the MR, and it is a key intermediate for hexose-catalyzed dehydration reactions under acidic conditions. As depicted in Figure 3a, both MCSJ and the model system consistently increased the 5-HMF content throughout the evaporation process. Specifically, the 5-HFM content in MCSJ significantly increased from 1.367 µg/g DS to 5.485 µg/g DS by 301.24%, which was consistent with the findings of Berk et al. (2019) [50]. The accumulation of 5-HMF in MCSJ was primarily ascribed to the intermediate stages of the MR, specifically through the 1,2-enolization pathway leading to the formation of 3-DG, which subsequently underwent further reactions to yield 5-HMF [51]. The notable increase in 5-HMF content during the 25–30 min period might be attributed to the rapid increase in 3-DG. The 5-HMF content within the model system increased at a stable rate, indicating that its MR was more stable than that of MCSJ throughout the evaporation process.

#### 3.4.3. Changes in CML and CEL

CML and CEL are both prominent markers of advanced glycation end products (AGEs) in the MR of foods, and they are also commonly used as key markers of the extent of MR [52]. The variations in CML and CEL concentrations within MCSJ and the model system during the evaporation process exhibited notable similarities. Initially, there was a gradual augmentation (within the first 20 min), followed by a rapid increase (Figure 3b,c). This progression was predominantly influenced by an elevation in the boiling point (i.e., the reaction temperature). Consequently, this enhanced the concentration levels within both MCSJ and the model system, which accelerated the MR reaction and then produced more CML and CEL. Notably, the contents of CML (0.174–0.222 µg/g DS) and CEL (0.122–0.180 µg/g DS) in MCJS were much higher than those of the model system. Simultaneously, the increase in CML (0.048 µg/g DS) and CEL (0.058 µg/g DS) in MCJS was higher than that in the model system (0.038 µg/g DS and 0.028 µg/g DS), indicating that the actual production rate of CML and CEL in MCJS exceeded that observed in the model systems during the evaporation process. One of the main reasons was that the precursor substances, such as 3-DG, MGO, and AGO in MCSJ, resulted from the MR during the MCSJ preparation process and continued to undergo the MR to produce CML and CEL. Meanwhile, the amount of CML and CEL produced by the MR during evaporation was affected by the reactant compositions and the coexisting substances [53]. In contrast, the amino acids and coexisting substances were quite different in MCJS and model systems. The MCJS contains 18 amino acids and various polyphenols, as well as metal ions originating from sugarcane in MCSJ, which are directly involved in the MR or influence the MR during the evaporation process [54,55], resulting in different generations of CML and CEL in MCSJ and the model systems [56].

#### 3.4.4. Changes in the Content of Melanoidins

Melanoidin is a dark-colored, high-molecular-weight complex compound with many nitrogen-containing groups, which is produced at the final stage of the MR [57]. As shown in Figure 3d, the initial content of melanoidins in MCSJ was up to 318.75 µg/g DS, providing further evidence that the MR between reducing sugars and amino acids already occurred during the MCSJ preparation process. During the evaporation process, the melanoidin content in MCSJ and the model system continued to increase linearly to 443.753 µg/g DS and 74.412 µg/g DS, respectively, which was very similar to the contents of CML and CEL, leading to a remarkable change in colorimetric parameters.

### 3.5. Kinetic Parameters of MR During Vacuum Evaporation

#### 3.5.1. Simple Kinetic Modeling

The kinetic parameters of the colorimetric parameters in the model systems under different temperatures are presented in Table 3. The zero-level, first-level, and second-level kinetic models could represent the change of L* and b* with an R^2^ of greater than 0.84, and the zero-level kinetic model best fits the change of a* and ΔE*. This indicated that the color difference of samples under different evaporation temperatures was linear over time. Simultaneously, the reaction constants (k) of a*, b*, and ΔE* exhibited a positive correlation with rising temperature, suggesting that the color difference in the model system increases as the evaporation temperature rises.

The apparent activation energies (E_a_) of L*, a*, b*, and ∆E* during the evaporation of model systems were 20.835 kJ/mol, 8.199 kJ/mol, 19.056 kJ/mol, and 19.87 kJ/mol, respectively, which was similar to the E_a_ of browning for beet juice concentrate stored in aluminium foil [58]. However, the E_a_ values were lower than those of the color difference for pineapple juice (36.85 kJ/mol–65.27 kJ/mol) reported in [59], indicating that the MR of the MCSJ model system was much more sensitive to temperature (60–90 °C). This may be related to the different chemical composition of the reactant system [60] (e.g., vitamin C, sugars, amino acids, etc.) and different reaction conditions.

The fitting results of the key products of MR in the model system under different temperatures are shown in Table 4. For MGO, 5-HMF, CML, and CEL, the first-level kinetic model provided the most appropriate fitting effect, suggesting that the logarithmic concentration of these products exhibits a linear relationship with the reaction time. The zero-level kinetic model of GO, 3-DG, and melanoidin had the best fitting effect, indicating that the concentration of GO, 3-DG, and melanoidin has a linear correlation with the reaction time.

A higher reaction rate constant (k) was observed for melanoidin, followed by 3-DG and MGO in descending order, indicating that the production of these products dominated the MR in the model system during evaporation. The kinetics model depicted further illustrates that the absolute values of the reaction constants (k) for MR products increased with temperature. Specifically, at 90 °C, the k values of GO, MGO, 3-DG, 5-HMF, CML, and melanoidins were 1.42, 1.69, 2.11, 1.82, 1.22, and 2.04 times higher than the values at 60 °C, indicating that the formation rate of the key MR products increased with higher reaction temperatures within an appropriate reaction time [61]. These findings emphasize the impact of varying reaction conditions on the rate of the MR. Moreover, the E_a_ values of GO, MGO, 3-DG, 5-HMF, CML, and melanoidins of the MCSJ model system during evaporation were determined to be 11.464 kJ/mol, 16.656 kJ/mol, 25.691 kJ/mol, 18.836 kJ/mol, 6.972 kJ/mol, and 22.276 kJ/mol, respectively. Notably, 3-DG exhibited the highest E_a_ value, suggesting that its generation was much more temperature-sensitive than the other MR products. The E_a_ of HMF was quite similar to that of 5-HMF (14.85 kJ/mol) in the glucose–asparagine–linoleic acid system [62] and was much lower than the Ea of 5-HMF and CML in the infant formula model (54.4 kJ/mol, 46.9 kJ/mol, respectively) discussed by Damjanovic et al. (2010) [63]. This discrepancy in E_a_ might be attributed to variations in these studies’ reaction systems and working conditions.

#### 3.5.2. Multiple-Response Model

**Figure 4:**
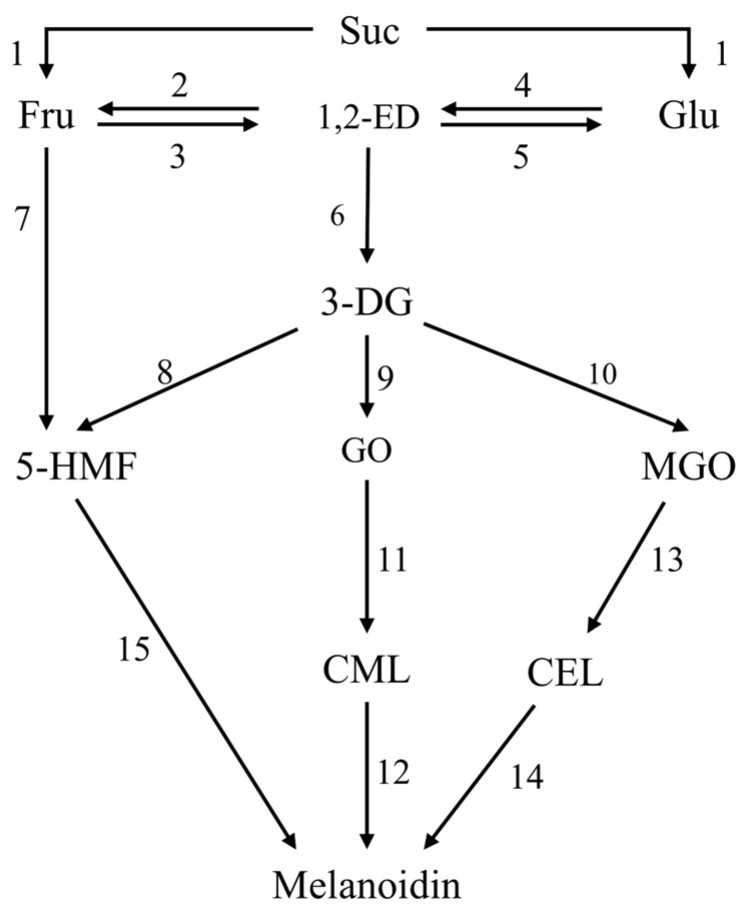
Comprehensive mechanism model of MR of model system during vacuum evaporation. Different numbers indicate different reaction pathways.

Referring to the general mechanism of the MR reported previously [64], the present study proposed a comprehensive model of the MR (Figure 4). The reaction rate constants of each reaction step were obtained by numerically integrating the differential equations and estimating the parameters using Athena Visual Studio (v.21.1) based on Equations (A1)–(A11) in Appendix A, and the goodness-of-fit of different models was evaluated based on R^2^. The kinetic rate constant (k_1_) for the hydrolysis of sucrose to glucose and fructose was approximated to be zero or virtually zero across all temperature ranges, indicating that the sucrose hydrolysis rate was uncertain, possibly because the glucoside bonds in sucrose easily cleaved to generate a fructofuran cation (FFC) at high temperatures [38], which affected its hydrolysis. However, the FFC was a very active intermediate that could not be detected experimentally. Therefore, its impact on k1 could not be evaluated, resulting in an uncertain reaction rate, which was in contrast to the relatively stable decrease in sugar content in the model system.

The isomerization rate constants of fructose-1,2-enediol (k_2_, k_3_) and glucose to 1,2-enediol (k_4_ and k_5_) are shown in Table 5. k_4_ at all temperatures and k_2_ at 70 °C and 80 °C could not be estimated within the 95% HPD. This was attributed to the failure to quantify the 1,2-enolation products, which were mainly generated by the interconversion of glucose and fructose through 1,2-enolization as a key intermediate during the initial phases of the MR, and these products subsequently underwent decomposition processes, ultimately yielding 3-deoxyglucosone (3-DG) with k_6_ (0.00343–0.0506 h^−1^) [65]. Thus, the presence of these unquantified compounds would impair the adequacy of the model’s fit to the experimental data and the reliability of the reaction rate determination. However, if the 1,2-enediol intermediates were excluded from the mathematical model, the simulation results were poorly fitted [38]. This suggested that the enolization of glucose and fructose was one key reaction pathway in MCSJ under vacuum evaporation. k_3_ and k_5_ were much higher than k_2_, and k_4_, indicating that the formation rate of 1,2-enolation products was much too higher than its consumption rate, and k_5_ was higher than k_3_ at 60 °C and 70 °C, while it was lower than k_3_ at 80 °C and 90 °C, indicated that fructoenolization is highly sensitive to temperature.

The formation pathways of 5-HMF in MCSJ included fructose dehydration (k_7_) and 3-DG dehydration (k_8_), and the k_8_ values (20,453–3899 h^−1^) under different temperatures were significantly higher than the k_7_ values (about 0.01 h^−1^), indicating that 5-HMF in MCSJ during evaporation was mainly generated by 3-DG dehydration, which was consistent with the findings of Sen et al. (2023) [66] and Tas et al. (2017) [67] that the contribution of fructose dehydration for 5-HMF production was much lower than that of the 3-DG pathway. However, this was in contrast to the findings of Gürsul et al. (2020) [46] that 5-HMF formation by fructose dehydration was two times higher than that of 3-DG dehydration in apple juice, orange juice, and peach blossom honey. This difference might be due to the different reaction substrates. At the same time, fructose could directly generate FFC without a heat-controlled ring-opening process because of its cyclic form and continue to produce 5-HMF, which also might affect fructose dehydration.

The formation pathways of short-chain α-dicarbonyl groups proposed in this study were as follows: long-chain α-dicarbonyl groups (3-DG) were formed from glucose and fructose through 1, 2-enolization, and then short-chain α-dicarbonyl groups, such as GO and MGO, were generated by the reverse aldehyde reaction of 3-DG [68,69]. The investigation demonstrated that the rate constant for the conversion of 3-DG (k_10_) to MGO was substantially greater than that for the conversion of GO (k_9_) to MGO, indicating that 3-DG was prone to generating MGO. Thus, the MGO contents were much higher than GO in the model systems during evaporation.

As the MR continued, α-dicarbonyl compounds underwent reactions with amino acids, leading to the formation of advanced glycosylation end products (AGEs), such as CML and CEL. CML and CEL are homologues produced by GO and MGO, respectively. The reaction rate constant of MGO to CEL (k_13_) was greater than that of GO to CML(k_11_), probably because the MGO content in the model system was greater than that of GO.

During the terminal phase of the MR, 5-HMF, CML, and CEL continued to react to produce the final product, melanoidins, and 5-HMF generated melanoidins at a much faster rate constant (k_15_) than CML(k_12_) and CEL(k_14_). The rate constants of CML to melanoidins at 80 °C and 90 °C and CEL to melanoidins at 70 °C and 90 °C were 0.001 h^−1^ and 0 h^−1^, respectively, indicating that these reactions occur at very low reaction rates compared with that of the 5-HMF. Therefore, 5-HMF to melanoidins was the main pathway for generating melanoidins in MCSJ during vacuum evaporation.

## 4. Conclusions

During the evaporation process, amino acids in both MCSJ and model systems react with sucrose, fructose, and glucose through the Maillard reaction, significantly affecting the system’s color. During the evaporation process of the MCSJ and the model system, both fructose and glucose showed a decreasing trend, while the color difference value, GO, MGO, 3-DG, 5-HMF, CML, and melanoidin content exhibited an upward trend as both the temperature and duration were increased. These variations followed straightforward kinetic models. The multiple-response model established revealed that the contents of glucose and fructose showed a decreasing trend despite the sucrose hydrolysis reaction, glucose isomerization to 1,2-en-diol was faster than fructose conversion, and 5-HMF was formed mainly through 3-DG dehydration reaction rather than the fructose pathway. MGO and GO were also formed by the degradation of 3-DG, of which the generation constant of MGO was much higher. 5-HMF, CML, and CEL continued to react to produce melanoidin, in which 5-HMF generates the melanoidin much faster than CML and CEL, so 5-HMF was the main pathway used to generate melanoidin. By monitoring the changes in the concentrations of Maillard reaction substrates and products and the color value variations, this study developed simple kinetic and multi-response kinetic models, enhancing our overall understanding of the Maillard reaction mechanisms and key pathways for sugar production. For the first time, it revealed the kinetics of the Maillard reaction in the membrane-filtered sugarcane juice (MCSJ) model system, providing theoretical guidance for controlling the color and specific Maillard reaction products in sugar products.

## Figures and Tables

**Table 1:** Main components and contents of the model system.

**Composition**	**Content (g/100 mL)**
Sucrose	12.475
Fructose	0.441
Glucose	0.514
Proline	0.45 × 10^−3^
Histidine	1.03 × 10^−3^
Lysine	0.56 × 10^−3^

**Table 2:** Content changes of amino acids and protein in MCSJ during vacuum evaporation.

mg/g DS	Heating Time (min)				
	0	10	20	25	30
Asp	0.4827 ± 0.0011 ^e^	0.5852 ± 0.0004 ^b^	0.6216 ± 0.0009 ^a^	0.5194 ± 0.0008 ^d^	0.5452 ± 0.0020 ^c^
Glu	3.0179 ± 0.0133 ^c^	3.3056 ± 0.0075 ^b^	3.4516 ± 0.0012 ^a^	2.8779 ± 0.0015 ^d^	3.0075 ± 0.0080 ^c^
Thr	0.0878 ± 0.0001 ^e^	0.41056 ± 0.0008 ^b^	0.1125 ± 0.0006 ^a^	0.0950 ± 0.0005 ^d^	0.0971 ± 0.0002 ^c^
Ser	0.2274 ± 0.0005 ^c^	0.2459 ± 0.0002 ^b^	0.2579 ± 0.0005 ^a^	0.2151 ± 0.0006 ^e^	0.2243 ± 0.0001 ^d^
Gly	0.0331 ± 0.0001 ^b^	0.0324 ± 0.0001 ^c^	0.0385 ± 0.0001 ^a^	0.0325 ± 0.0002 ^c^	0.0322 ± 0.0002 ^d^
Ala	0.2745 ± 0.0011 ^d^	0.3042 ± 0.0010 ^b^	0.3204 ± 0.0052 ^a^	0.2675 ± 0.0003 ^e^	0.2788 ± 0.0001 ^c^
Cys	0.0120 ± 0.0002 ^a^	0.0764 ± 0.0886 ^b^	0.0177 ± 0.0004 ^a^	0.0143 ± 0.0003 ^a^	0.0137 ± 0.0006 ^a^
Val	0.1245 ± 0.0032 ^e^	0.1508 ± 0.0032 ^b^	0.1570 ± 0.0012 ^a^	0.1318 ± 0.0002 ^d^	0.1367 ± 0.0004 ^c^
Met	0.0113 ± 0.0003 ^c^	0.0229 ± 0.0082 ^a^	0.0200 ± 0.0021 ^ab^	0.0176 ± 0.0002 ^b^	0.0175 ± 0.0002 ^b^
Ile	0.0543 ± 0.0003 ^c^	0.0768 ± 0.0063 ^a^	0.0771 ± 0.0002 ^a^	0.0637 ± 0.0031 ^a^	0.0665 ± 0.0010 ^b^
Leu	0.0343 ± 0.0001 ^e^	0.0521 ± 0.0018 ^b^	0.0534 ± 0.0046 ^a^	0.0443 ± 0.0004 ^d^	0.0464 ± 0.0021 ^c^
Tyr	0.0435 ± 0.0045 ^d^	0.0626 ± 0.0007 ^ab^	0.0684 ± 0.0017 ^a^	0.0539 ± 0.0088 ^c^	0.0615 ± 0.0005 ^b^
Phe	0.0276 ± 0.0017 ^c^	0.0297 ± 0.0007 ^bc^	0.0313 ± 0.0002 ^b^	0.0349 ± 0.0011 ^a^	0.0316 ± 0.0016 ^b^
Hypro	0.2699 ± 0.0046 ^c^	0.2919 ± 0.0058 ^b^	0.3118 ± 0.0040 ^a^	0.2635 ± 0.0015 ^d^	0.2705 ± 0.0012 ^c^
Pro	0.0302 ± 0.0036 ^d^	0.0519 ± 0.0010 ^b^	0.0625 ± 0.0029 ^a^	0.0438 ± 0.0118 ^c^	0.0519 ± 0.0013 ^b^
Total amino acids	4.8844 ± 0.0285 ^d^	5.5674 ± 0.0519 ^b^	5.7911 ± 0.0073 ^a^	4.8118 ± 0.0217 ^e^	5.0261 ± 0.0071 ^c^
Protein	1.3190 ± 0.0060 ^a^	1.2450 ± 0.0310 ^b^	1.0320 ± 0.0100 ^c^	0.8910 ± 0.0180 ^d^	0.8130 ± 0.0070 ^e^

Note: Different letters indicate significant differences (*p* < 0.05).

**Table 3:** Kinetic parameters of colorimetric parameters in model system.

Temperature (°C)	Kinetic Model	Indicator	k (h^−1^)	R^2^	E_a_ (kJ/mol)	Indicator	k (h^−1^)	R^2^	E_a_ (kJ/mol)
60	0	L*	−1.5060	0.898	20.835 ± 0.740	a*	0.4104	0.815	8.199 ± 2.883
	1		−0.0415	0.897			--	--	
	2		0.0012	0.889			--	--	
70	0		−1.6950	0.928			0.4926	0.943	
	1		−0.0467	0.925			--	--	
	2		0.0013	0.93			--	--	
80	0		−1.8702	0.952			0.5292	0.977	
	1		−0.0515	0.950			--	--	
	2		0.0014	0.947			--	--	
90	0		−2.9322	0.985			0.5244	0.882	
	1		−0.0816	0.984			--	--	
	2		0.0023	0.981			--	--	
60	0	b*	0.8292	0.841	19.056 ± 8.767	ΔE*	1.7478	0.883	19.867 ± 8.767
	1		0.8196	0.862			--	--	
	2		−0.8214	0.905			--	--	
70	0		1.4604	0.942			2.3016	0.937	
	1		1.272	0.973			--	--	
	2		−1.1406	0.984			--	--	
80	0		1.4526	0.985			2.3916	0.973	
	1		1.2486	0.964			--	--	
	2		−1.107	0.922			--	--	
90	0		1.5474	0.993			3.3378	0.996	
	1		1.3134	0.972			--	--	
	2		−1.1514	0.931			--	--	

-- means the number cannot be calculated.

**Table 4:** Kinetic parameters of key MR products in model system.

Temperature (°C)	Types	Indicator	k (h^−1^)	R^2^	E_a_ (kJ/mol)	Indicator	k (h^−1^)	R^2^	E_a_ (kJ/mol)
60	0	GO	0.1476	0.972	11.464 ± 1.414	MGO	0.5694	0.814	16.656 ± 2.725
	1		5.1860	0.961			7.1154	0.974	
	2		−293.1504	0.754			−186.4026	0.899	
70	0		0.1644	0.976			0.6654	0.841	
	1		5.3688	0.951			7.1286	0.994	
	2		−293.4474	0.731			−173.9076	0.767	
80	0		0.1770	0.907			0.7308	0.794	
	1		5.5398	0.980			7.4682	0.993	
	2		−297.0744	0.768			−179.5134	0.831	
90	0		0.2106	0.937			0.9624	0.688	
	1		5.9041	0.974			7.8924	0.998	
	2		−300.5466	0.753			−176.8578	0.793	
60	0	3-DG	11.5734	0.838	25.691 ± 3.081	5-HMF	0.1158	0.812	18.836 ± 5.755
	1		10.9242	0.946			5.0670	0.991	
	2		−71.6022	0.562			−350.2164	0.845	
70	0		13.4322	0.924			0.1794	0.917	
	1		10.5924	0.816			6.0372	0.980	
	2		−70.4520	0.515			−362.4492	0.811	
80	0		18.7362	0.959			0.1908	0.892	
	1		11.178	0.777			6.1572	0.987	
	2		−70.4040	0.508			−362.5248	0.808	
90	0		24.4548	0.960			0.2088	0.883	
	1		11.4942	0.737			6.3372	0.990	
	2		−70.3302	0.504			−363.7416	0.806	
60	0	CML	0.0612	0.816	6.972 ± 0.621	CEL	0.0542	0.830	11.136 ± 0.668
	1		7.9032	0.994			7.9440	0.993	
	2		−2692.6134	0.769			−3124.0776	0.751	
70	0		0.0642	0.830			0.0593	0.880	
	1		7.8348	0.989			8.0424	0.978	
	2		−2622.9192	0.701			−3073.7130	0.699	
80	0		0.0708	0.841			0.0678	0.867	
	1		7.7832	0.958			8.1606	0.972	
	2		−2557.2288	0.625			−3022.2282	0.648	
90	0		0.0744	0.844			0.0750	0.854	
	1		7.8954	0.958			8.4744	0.981	
	2		−2559.3384	0.623	22.353 ± 6.875		−3057.0618	0.672	
60	0	Mela-noid	69.5022	0.970					
	1		4.1634	0.948					
	2		−0.351	0.750					
70	0		116.7768	0.942					
	1		4.9932	0.908					
	2		−0.3594	0.662					
80	0		124.6932	0.972					
	1		5.0064	0.797					
	2		−0.3552	0.586					
90	0		141.6066	0.977					
	1		5.2716	0.840					
	2		−0.3595	0.602					

**Table 5:** The reaction rate constant and activation energy of the kinetic model in the 95% maximum posterior density (HPD) range.

Elementary Reaction Steps		60 °C		70 °C		80 °C		90 °C	
		k (h^−1^)	HPD	k (h^−1^)	HPD	k (h^−1^)	HPD	k (h^−1^)	HPD
k_1_	Suc → Glu+ Fru	0.0000	*ind	0.0000	*ind	0.0087	±0.0070	0.0000	*ind
k_2_	1,2-E, D → Fru	0.0031	±0.0634	0.0000	*ind	0.0000	*ind	0.0246	±0.0736
k_3_	Fru → 1,2- E, D	0.0385	±0.0915	0.0352	±0.0112	1.8862	±0.4520	0.0732	±0.0736
k_4_	1,2- E, D → Glu	0.0001	*ind	0.0001	*ind	0.0001	*ind	0.0001	*ind
k_5_	Glu → 1,2- E, D	0.0548	±0.0880	0.0377	±0.0382	0.6368	±0.5970	0.0587	±0.0707
k_6_	1,2-E, D→3-DG	0.0445	±0.0375	0.0343	±0.0196	0.5366	±0.3574	0.0506	±0.0252
k_7_	Fru → 5-HMF	0.0100	±0.0001	0.0100	±0.0001	0.0149	±0.0149	0.0100	±0.0001
k_8_	3-DG→ 5-HMF	38,991	±37,170	20,453	±10,810	27,521	±21,480	28,918	±22,910
k_9_	3-DG → GO	0.2808	±0.1528	0.2330	±0.1109	0.2180	±0.1239	0.2940	±0.2006
k_10_	3-DG → MGO	46.4663	±29.7000	46.6424	±21.4401	51.9015	±29.9000	56.3724	±35.2000
k_11_	GO → CML	0.0079	±0.0017	0.0073	±0.0012	0.0065	±0.0036	0.0069	±0.0021
k_12_	CML → melanoidin	0.0100	±0.0001	0.0100	±0.0010	0.0010	±0.0010	0.0010	±0.0010
k_13_	MGO → CEL	114.1332	±87.4500	147.5678	±80.5500	167.6380	±116.4000	172.7986	±120.0400
k_14_	CEL →melanoidin	0.0050	±0.0297	0.0000	±0.0001	0.0043	±0.0020	0.0000	±0.0000
k_15_	5-HMF →melanoidin	31,945	±26,290	23,069	±12,100	31,232	±24,990	28,874	±19,900

*ind—indeterminate, which means there is significant uncertainty in the estimated parameter within 95% confidence interval.

## Data Availability

The original contributions presented in the study are included in the article; further inquiries can be directed to the corresponding authors.

## Abbreviations

The following abbreviations are used in this manuscript:

MCSJ	Membrane-clarified sugarcane juice.
MR	Maillard reaction.
GO	Glyoxal.
MGO	Methyl glyoxal.
3-DG	3-deoxyglucosaldoketone.
5-HMF	5-hydroxymethylfurfural.
CML	Carboxymethyl Lysine.
CEL	Carboxyethyl Lysine.
Lys	Lysine.
His	Histidine.
Pro	Proline.

## Appendix A

The Supplementary formula to this article is as follows:

(A1) d[Suc]/dt = − k_1_ [Suc]

(A2) d[Fru]/dt = k_1_ [Suc] + k_2_ [1,2-ED] − k_3_ [fru]

(A3) d[1,2-ED]/dt = k_3_ [Fru] + k_5_ [Glu] − (k_2_ + k_4_ + k_6_) [1,2-ED]

(A4) d[Glu]/dt = k_1_ [Suc] + k_4_ [1,2-ED] − k_5_ [Glu]

(A5) d[3-DG]/dt = k_6_ [1,2-ED] − (k_8_ + k_9_ + k_10_) [3-DG]

(A6) d[5-HMF]/dt = k_7_ [Fru] + k_8_ [3-DG] − k_15_ [5-HMF]

(A7) d[GO]/dt = k_9_ [3-DG] − k_11_ [GO]

(A8) d[MGO]/dt = k_10_ [3-DG] − k_13_ [MGO]

(A9) d[CML]/dt = k_11_ [GO] − k_12_ [CML]

(A10) d[CEL]/dt = k_13_ [MGO] − k_14_ [CEL]

(A11) d[melanoidin]/dt = k_12_ [CML] + k_14_ [CEL] + k_15_ [5-HMF]
